# Flexible Mixed-Potential-Type (MPT) NO_2_ Sensor Based on An Ultra-Thin Ceramic Film

**DOI:** 10.3390/s17081740

**Published:** 2017-07-29

**Authors:** Rui You, Gaoshan Jing, Hongyan Yu, Tianhong Cui

**Affiliations:** 1 State Key Laboratory of Precision Measurement Technology and Instruments, Tsinghua University, Beijing 100084, China; your13@mails.tsinghua.edu.cn (R.Y.); yuhongyan@mail.tsinghua.edu.cn (H.Y.); 2 Department of Precision Instrument, Tsinghua University, Beijing 100084, China; 3 Department of Mechanical Engineering, University of Minnesota, Minneapolis, MN 55455, USA

**Keywords:** flexible mixed-potential-type (MPT) sensor, ultra-thin ceramic film, mesoporous TiO_2_

## Abstract

A novel flexible mixed-potential-type (MPT) sensor was designed and fabricated for NO_2_ detection from 0 to 500 ppm at 200 °C. An ultra-thin Y_2_O_3_-doped ZrO_2_ (YSZ) ceramic film 20 µm thick was sandwiched between a heating electrode and reference/sensing electrodes. The heating electrode was fabricated by a conventional lift-off process, while the porous reference and the sensing electrodes were fabricated by a two-step patterning method using shadow masks. The sensor’s sensitivity is achieved as 58.4 mV/decade at the working temperature of 200 °C, as well as a detection limit of 26.7 ppm and small response time of less than 10 s at 200 ppm. Additionally, the flexible MPT sensor demonstrates superior mechanical stability after bending over 50 times due to the mechanical stability of the YSZ ceramic film. This simply structured, but highly reliable flexible MPT NO_2_ sensor may lead to wide application in the automobile industry for vehicle emission systems to reduce NO_2_ emissions and improve fuel efficiency.

## 1. Introduction

Air pollution from car exhaust has caused plenty of environmental disasters in several countries and affected a large number of lives around the world due to the rapid development of the automobile industry [1,2]. In particular, nitrogen oxides gases (NO/NO_2_), as harmful gas pollutants, cause a variety of adverse health issues such as bronchitis, serious neurodegenerative diseases, and cancers [3]. These toxic gases also lead to urban environmental pollution issues such as acid rain and chemical smog [4]. To accurately and precisely monitor the NO_X_ gases, especially NO_2_, from car exhaust in a harsh working environment with a high temperature and large vibration, a high-performance NO_2_ sensor was in high demand for vehicle emission systems to reduce NO_2_ emissions and improve vehicle fuel efficiency. 

A few NO_2_ sensors have been developed based on various sensing techniques, including metal oxide semiconductor sensing [5,6], infrared light absorption [7,8] and electrochemical reaction [9,10,11]. Among these sensors, one type of electrochemical NO_2_ sensor, mixed-potential-type (MPT) NO_2_ sensor based on Y_2_O_3_-doped ZrO_2_ (YSZ) solid electrolyte material, is widely investigated for a vehicle emission systems due to the sensor’s excellent chemical and thermal stability at high working temperatures [12,13]. A typical MPT NO_2_ sensor includes several functional layers: a bulk substrate; a heating electrode layer to let the sensor working at an appropriate temperature; a YSZ solid electrolyte layer; a reference electrode layer, and; a sensing electrode layer on top of the YSZ layer [14,15]. NO_2_ gases’ reaction with oxygen in the ambient environment occurs at the triple-phase-boundary (TPB) interface between the YSZ solid electrolyte layer and the sensing electrode layer. Eventually, the electrochemical potential difference between the reference electrode layer and the sensing electrode layer is measured and correlated with the NO_2_ gases concentration [16,17]. 

The MPT NO_2_ sensor based on various sensing electrode materials has been developed to detect NO_2_ gas. A composite material of In_2_O_3_ and MoO_3_ has been utilized as the sensing material for NO_2_ detection. With the MoO_3_ doping ratio optimized at 5 wt%, the largest sensitivity of 59 mV/decade for NO_2_ was achieved in the range of 10–200 ppm at 500 °C [18]. Another new type of composite oxide Co_3_V_2_O_8_ mixed with YSZ particles was used as the NO_2_ gases sensing material for an MPT sensor. With the mixture ratio between Co_3_V_2_O_8_ mixed and YSZ particles configured at 60:40, the largest sensitivity of 81 mV/decade for NO_2_ was achieved in the range of 10–400 ppm at 700 °C [19]. To further increase MPT sensors’ sensitivity, various techniques have been applied to increase the sensing material’s area, including etching the YSZ substrate surface using the hydrofluoric acid and template method with a self-assembled polystyrene sphere [20,21]. Due to the complexity of an MPT NO_2_ sensor, several crucial steps to fabricate an MPT NO_2_ sensor are realized manually and greatly affect the sensor’s reliability and uniformity. In particular, the YSZ solid electrolyte layer needs to be fabricated as a functional layer with the thickness delicately controlled on a bulk substrate for all of these sensors. Moreover, an MPT sensor is usually placed close to a car engine and its working temperature is well above 600 °C, with high power consumption and a low shelf life. While it is also desirable to install an MPT sensor inside a low-temperature region of an exhaust pipe (100–300 °C) to monitor NO_2_ emissions, such a disadvantage greatly limits the application of conventional MPT NO_2_ sensors.

**Figure 1:**
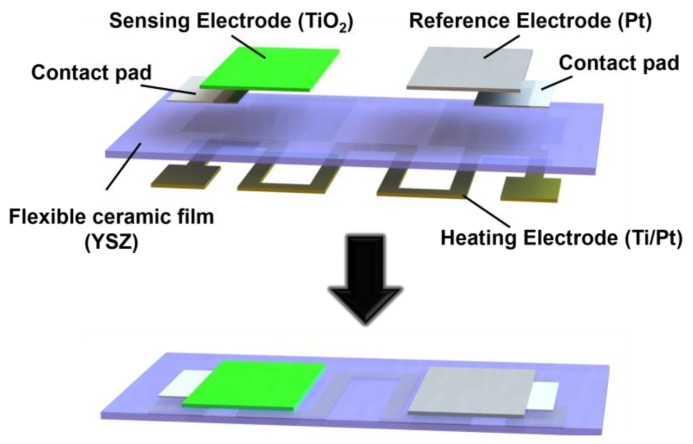
Schematic diagram of a flexible mixed-potential-type (MPT) NO_2_ sensor.

In this paper, a novel flexible mixed-potential-type (MPT) sensor based on an ultra-thin ceramic film was developed and utilized to detect NO_2_. As shown in Figure 1, the sensor is composed of a heating electrode, a reference electrode, an NO_2_ sensing electrode on the back side and front side of an ultra-thin 3 mol% Y_2_O_3_-doped ZrO_2_ (YSZ-3Y) film 20 µm thick, respectively. The heating electrode was fabricated by a conventional lift-off process, while the porous reference and sensing electrodes were fabricated by a two-step patterning method using shadow masks. Due to the ceramic film’s low thermal conductivity and thin film thickness, a low power assumption of 1025 mW was consumed to reach 246 °C in an area of 8.0 mm by 6.4 mm. The sensor’s sensitivity could be achieved as 58.4 mV/decade at a working temperature of 200 °C. Additionally, the flexible sensor demonstrates superior mechanical stability after bending over 50 times due to the mechanical stability of the YSZ ceramic film. This flexible mixed-potential-type (MPT) sensor could be installed on an exhaust pipe’s circular surface without interfering with exhaust emission and could monitor the NO_2_ emission in a low-temperature region from 100 °C to 300 °C. This simple structured flexible MPT NO_2_ sensor fabricated by highly reliable methods exhibits excellent performance to detect NO_2_, which may lead to wide application in the automobile industry for vehicle emission systems to reduce vehicle emissions and improve fuel efficiency. 

## 2. Materials and Methods

### 2.1. Sensor Design and Simulation

**Figure 2:**
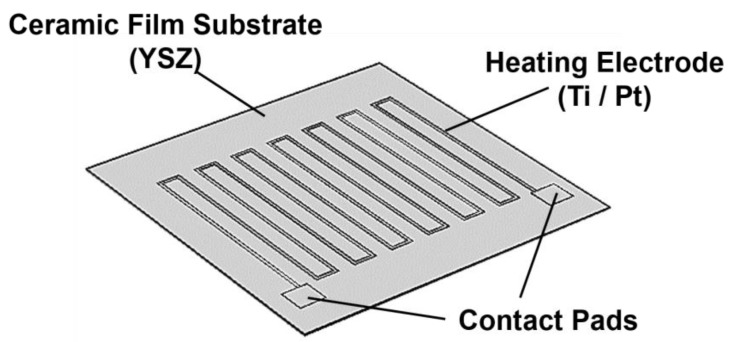
Geometry model of the sensor’s heating electrode for stimulation by COMSOL Multiphysics.

The MPT NO_2_ sensor’s heating electrode was designed and its electrothermal performance was simulated by COMSOL Multi-physics software (COMSOL Inc., Los Angeles, CA, USA). The heating electrode’s schematic diagram is illustrated in Figure 2 with two components: a YSZ based ultra-thin flexible ceramics film 20 µm thick, and; a Ti/Pt heating electrode. The heating electrode’s key design parameters are listed in Table 1, the heating electrode’s length is 94.8 mm with a width of 100 µm and a thickness of 4500 Å (500 Å Ti/ 4000 Å Pt). An adjustable driving voltage at 1 V–35 V was applied to the contact pads to investigate the electrothermal response of the heating electrode. In the heat transfer process, the ambient environment temperature was configured as 26 °C. It should be noted that the thermal conduction coefficient of the ceramic thin film is 2.7 m^−1^·K^−1^ according to technical information provided by the flexible YSZ ceramic film’s manufacturer [22], while Ti and Pt’s material parameters are from the COMSOL library.

### 2.2. Device Fabrication

The NO_2_ sensor was fabricated on a planar yttria-stabilized zirconia (YSZ) ceramic thin film (EnrG Inc., Buffalo, NY, USA) with two major processes. One is a lift-off process to fabricate a heating electrode, and the other is a two-step patterning process using shadow masks to fabricate the sensing and reference electrodes. The YSZ ceramic thin film was sandwiched between the heating electrode on the backside of the film and reference/sensing electrodes on the front side of the film.

**Figure 3:**
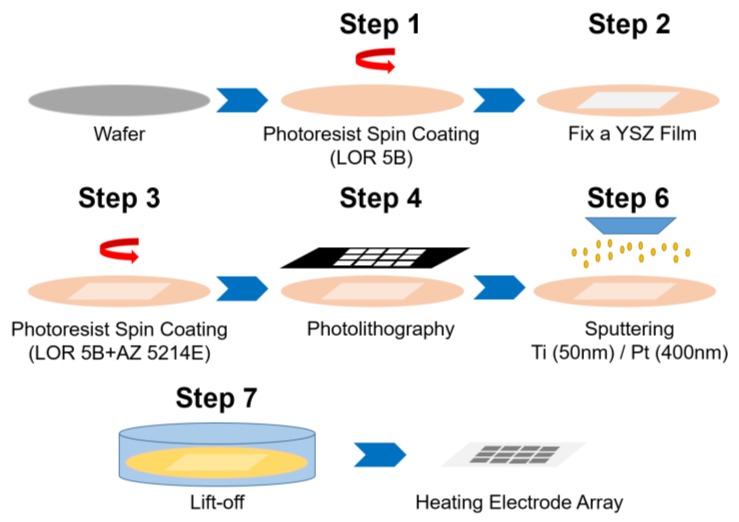
Schematic diagram of the fabrication process for the sensor’s heating electrode.

As shown in Figure 3, the heating electrode was fabricated by a lift-off process. First, a thin layer of photoresist (1.6 µm, LOR 5B, Microchem Corp., Westborough, MA, USA) was spin coated on a four-inch silicon wafer. Then the YSZ ceramic film was positioned on the wafer surface guided by a laser marker and fixed on the silicon substrate by drying the photoresist at 190 °C for 180 s. Next, the second and third layers of photoresist (1.6 µm, LOR 5B, Microchem Corp., Westborough, MA, USA; 1.4 µm, AZ5214e, MicroResist, Berlin, Germany) were deposited on the fixed YSZ film surface by spin coating sequentially and soft baked at 190 °C and 105 °C for 180 s, respectively. The photoresist films were exposed to UV light for 6 s using a mask aligner and developed for 90 s (MA150e, SUSS, MicroTec, Garching, Germany). Sequentially, 500 Å titanium and 4000 Å platinum metal layers were deposited by sputtering (904i, KDF Inc., Torrance, NJ, USA).

**Figure 4:**
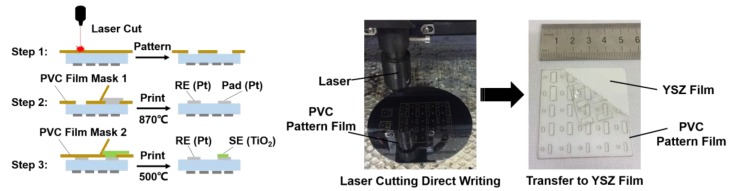
Schematic diagram of the fabrication process for the sensor’s reference and sensing electrodes.

Once the heating electrode was fabricated, the YSZ ceramic film with the heating electrode was flipped over and the reference electrode and the sensing electrode were fabricated on the front side of the film by a two-step patterning process using shadow masks, as shown in Figure 4. Thin and flexible PVC (Polyvinyl chloride) films were utilized as shadow masks for patterning the reference and sensing electrodes. First, PVC films were patterned by a laser cutter. Then the first PVC film was rolled and attached on the YSZ ceramic surface manually without generating bubbles aided by the electrostatic adhesion between the PVC film and YSZ substrate. The Pt reference electrode (RE) (2.0 mm × 6.0 mm) and the square-shaped platinum pad (1.0 mm × 2.0 mm) on the YSZ film were patterned by printing manually using platinum paste (Sino-platinum Metals Co., KunMing, China.). Next, the PVC film was removed and the film with Pt paste pattern was put on a hotplate at 150 °C for 10 min to cure the paste. Then, it was sintered at 870 °C for 1 h (P330, Nabertherm GmbH, Bremen, Germany). To fabricate the sensing electrode, an oxide solutions with 14 wt% mass fraction of TiO_2_ nanoparticles (diameter: 15 nm, Sigma Aldrich, MO, USA) were mixed up with anhydrous ethanol (99.7% purity, Sigma Aldrich, MO, USA) by magnetic stirring and acentric mixing process for 30 min. The second PVC film mask was used to pattern sensing electrode and mask the rest of the YSZ thin film surface. The TiO_2_ nanoparticles solution was dropped onto the sensing electrode area (2.0 mm × 6.0 mm). Eventually, a thin film of 0.5 µm TiO_2_ sensing film formed and was sintered at 500 °C for 30 min.

### 2.3. Electrical, Thermal, Surface Properties Characterization

**Figure 5:**
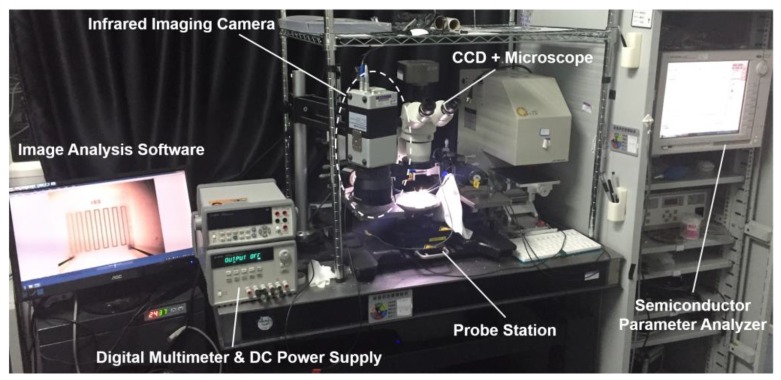
Testing platform for characterization of the sensor’s electrothermal properties, including a digital multimeter, a DC power supply, an infrared imaging camera, a probe station and a semiconductor parameter analyzer.

Electrical and thermal properties of the NO_2_ sensor were measured by a customized testing platform, as shown in Figure 5, including a digital multimeter, a DC power supply, an infrared imaging camera (Infra Tech VC HD Head 880, InfraTec, Dresden, Germany), a probe station and a semiconductor parameter analyzer (B1500A, Agilent, Santa Clara, CA, USA). The surface temperature profile of the NO_2_ sensor was obtained and analyzed by the infrared image analyzing software (IRBIS3, InfraTec, Dresden, Germany). Surface properties of the sensor were characterized by scanning electron microscopy (Quanta 200, FEI, Hillsboro, OR, USA), with a detector enabling energy-dispersive X-ray spectroscopy analysis to characterize the thin film material for reference and sensing electrodes. Finally, the raw data will be processed by OriginLab software.

### 2.4. Sensing Performance Characterization

**Figure 6:**
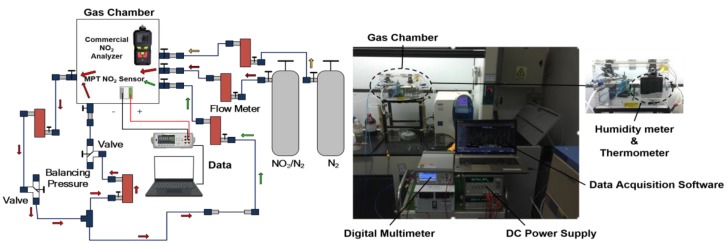
Testing platform for measuring the sensor’s gas sensing performance.

The NO_2_ gas sensing measurement was performed in a customized gas sensing and measurement platform. As shown in Figure 6, the testing system consists of a gas distribution module (0.15% NO_2_/99.85% N_2_), a gas chamber (15 L volume, humidity, flow rate adjustable), a detection and calibration system (PM80-X2, Eranntex, ShenZhen, China), and a data acquisition system (34461A Digit multimeter, Agilent, Santa Clara, CA, USA). The NO_2_ sensor was powered by a DC power supply (E3633A, Agilent, Santa Clara, CA, USA). A digital thermocouple was used to monitor the surface temperature of the device in real time. Gas sensing experiment was carried out in a clean room with the humidity of 13% and environment temperature of 21.2 °C inside the gas chamber, with an oxygen concentration of 20.8% in a standard atmosphere. 

## 3. Results and Discussion

**Figure 7:**
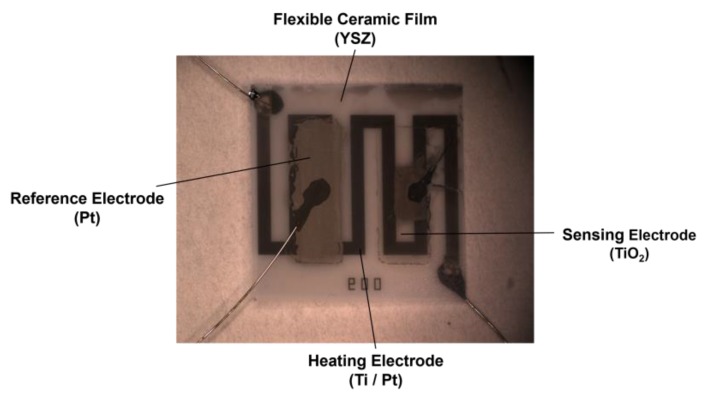
Optical image of a mixed-potential-type (MPT) sensor with a heating electrode, a reference electrode, an NO_2_ sensing electrode on the back side and front side of a ultra-thin Y_2_O_3_-doped ZrO_2_ (YSZ) ceramic film 20 µm thick.

A single NO_2_ sensor based on a flexible YSZ ceramic film was shown in Figure 7, with the Pt reference electrode and the TiO_2_ sensing electrode fabricated on the front side of the thin film, as well as the Ti/Pt heating electrode of 8.0 mm by 6.4 mm fabricated on the backside of the thin film. The heating electrode’s width is measured as 102.2 µm on average, and the thickness of the heating electrode is measured as 4200 Å. As for the Pt reference electrode, its dimension is 2.1 mm by 5.9 mm, while TiO_2_ sensing electrode’s dimension is 2.2 mm by 6.1 mm. 

**Figure 8:**
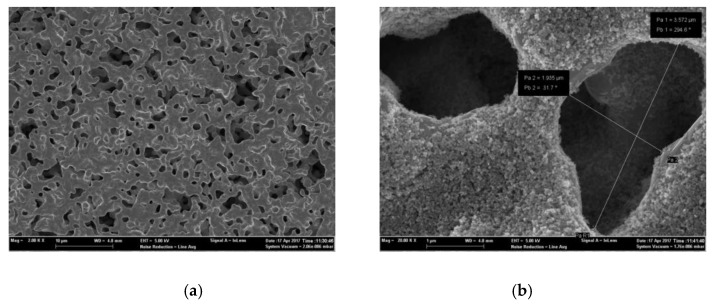

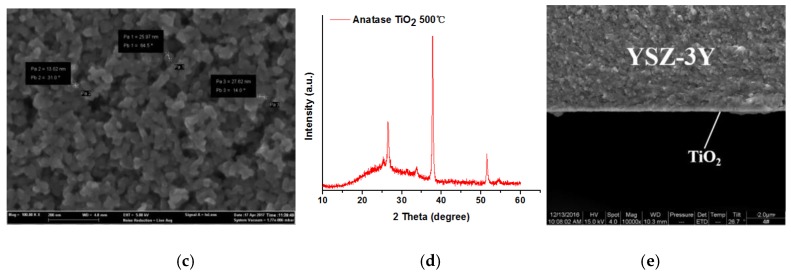
Scanning electron microscopy (SEM) image of (**a**) platinum reference electrode surface profile; (**b**) and (**c**) TiO_2_ sensing electrode surface profile at 20,000 and 100,000 magnification; (**d**) X-ray diffraction (XRD) profile of anatase TiO_2_ nanoparticles sintered at 500 °C; (**e**) SEM image of a cross section of YSZ thin film with the TiO_2_ sensing material.

The reference and sensing electrode surface profiles were characterized by scanning electron microscopy (SEM). As shown in Figure 8a, a porous Pt reference electrode was achieved by the shadow mask patterning method. Such a porous Pt surface with a porous size about several hundred nanometers will enable the rapid diffusion and permeation of oxygen and NO_2_ gases on the electrode surface. For the sensing electrode, a mesoporous layer consisting of TiO_2_ nanoparticles with a particle size of 10–20 nm formed after sintering treatment at 500 °C shown in Figure 8b and 8c. For the sensing electrode, homogenous anatase structured TiO_2_ nanoparticles were achieved for NO_2_ sensing as shown in Figure 8d. The thickness of the mesoporous TiO_2_ layer is about 0.5 µm, as shown in Figure 8e. These uniform nanometer-sized pores will increase the contact area and absorption between NO_2_ gas and the TiO_2_ sensing material.

### 3.1. Electrothermal Properties of the NO_2_ Sensor

The heating electrode’s resistance is measured as 378 Ω in good accordance with the simulated result of 333 Ω. Comparing to the simulation result, an increase of the heating electrode’s resistance is due to two factors: one is the decreased thickness of Ti/Pt film (4200 Å vs 4500 Å); the other is that thin film resistivity is typically higher than the material’s bulk resistivity [23]. 

**Figure 9:**
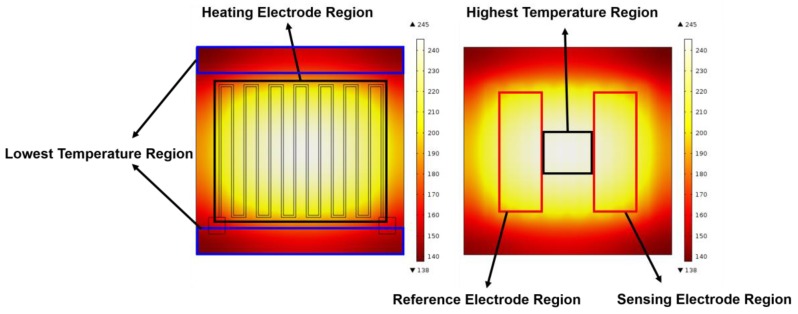
Surface temperature gradient distribution of the heating electrode by COMSOL simulation.

Simulated electrothermal properties of the NO_2_ sensor and temperature profile on the thin YSZ film was demonstrated in Figure 9. Due to the low thermal conduction coefficient of the ceramic thin film (2.7 m^−1^·K^−1^), the temperature profile is not perfectly uniform inside the heating electrode region. Therefore, five regions of the NO_2_ sensor were selected to investigate the electrothermal properties of the NO_2_ sensor: (1) highest temperature region in the center; (2) reference electrode region; (3) sensing electrode region; and the two lowest temperature regions outside of the heating electrode.

**Figure 10:**
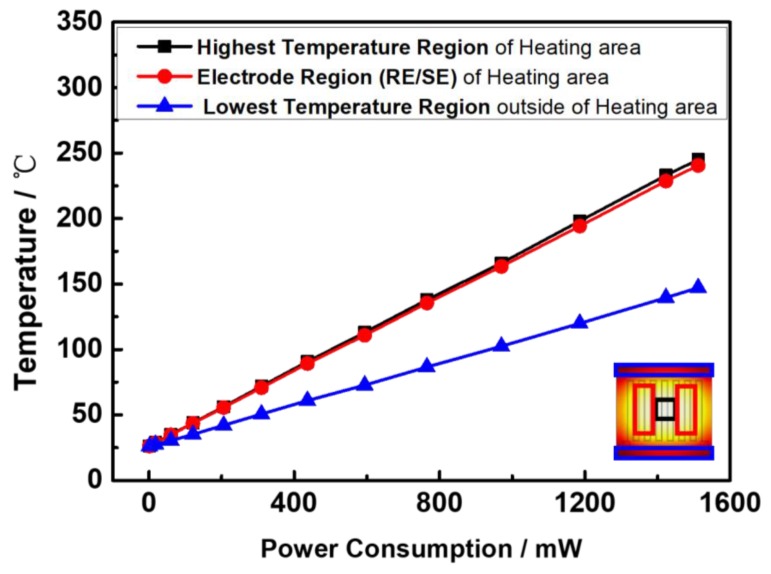
Working temperature vs. power consumption of the sensor by COMSOL simulation.

From the simulation results, as shown in Figure 10, it is derived and demonstrated that a uniform heating area is largely concentrated in the heating electrode area. To achieve the highest temperature of 245 °C in an area of 2.0 mm by 2.0 mm at the center of the heating electrode, the power consumption should be about 1512 mW. In the highest temperature region of 245 °C, the average temperate inside the heating electrode region is 214 °C. Outside the heating electrode, the average minimum temperature drops to 140 °C.

**Figure 11:**
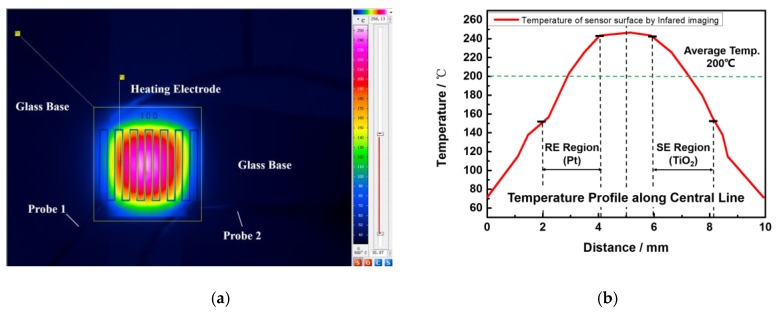
Testing results of the sensor’s surface temperature profile by the high-resolution infrared camera. (**a**) The sensor with an area of 10 mm × 10 mm with an applied voltage of 25 V; (**b**) Comparison of temperature profile along the sensor surface.

This phenomenon is verified by the corresponding experimental results in Figure 11. The temperature profile of the flexible NO_2_ sensor measured by the infrared thermal imaging. To achieve the highest temperature of 246 °C in the center of the electrode (an area of 2.6 mm by 2.6 mm), the power consumption is about 1025 mW, in good accordance with the simulation results. The highest temperature of 246 °C was obtained by a heating power of 1025 mW. The average temperature inside the heating electrode region is 173 °C. Inside the reference and sensing electrode regions, the temperature range is from 150 °C to 240 °C. Outside the heating electrode region, the average temperature dropped to 110 °C and the minimum temperature of the sensor is 70 °C. 

Comparing to our previous publications [24], power consumption is over 170 mW for an area of 0.36 mm^2^ on a quartz substrate, and 1105 mW for an area of 0.36 mm^2^ on a silicon substrate. Power assumption density of the flexible NO_2_ sensor, 20 mW/mm^2^, is about 1/20 of the power assumption density of the thin quartz gas sensor and 1/130 of the power assumption density of the bulk silicon gas sensor. Low power consumption density is due to two factors: low thermal conductivity of YSZ thin film and ultra-thin thickness of the film. The thermal conductivity of YSZ thin film is 2.7 W·m^−1^·K^−1^, close to that of quartz material (1.2 W·m^−1^·K^−1^) and much smaller than that of silicon material (149 W·m^−1^·K^−1^). As for the thin film thickness, it is 20 microns and much smaller than those of the thin quartz wafer (100 µm) and the silicon wafer (500 µm) we used in our previous publication [25]. Low thermal conductivity with thin film thickness results in a low thermal mass of the ceramic film and leads to smaller power consumption density of the flexible NO_2_ sensor.

### 3.2. NO_2_ Sensing Performances

**Figure 12:**
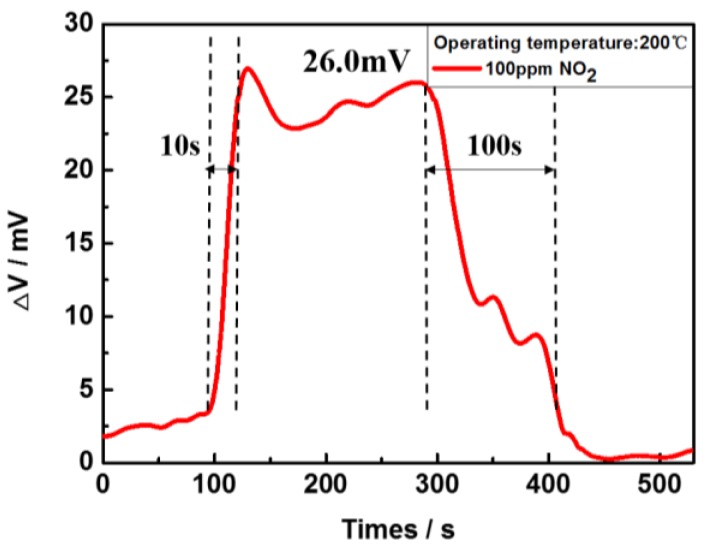
NO_2_ sensing response for the flexible YSZ-3Y based sensor to the concentration of 100 ppm NO_2_ at 200 °C.

The sensor’s response to NO_2_ gas is demonstrated in Figure 12. Once the sensor was heated to a working temperature of 200 °C, 100 ppm NO_2_ gas was delivered into the gas chamber. Then an electrochemical potential between the sensing electrode and the reference electrode was obtained as 26.0 mV. The sensor’s response and recovery time are 10 s and 100 s, respectively. Comparing to existing publications [10,26], the working temperature of the flexible gas sensor is 200 °C smaller than most of the publications, and may be due to uniform and concentrated heating region resulted from the ultra-thin ceramic film, as well as the uniform porous reference and sensing electrodes surface [27]. It should be noted that there may exist an optimal working temperature for the sensor’s sensitivity [28,29]. Higher temperature will lead to higher electrochemical potential induced by NO_2_ reaction, as well as the electrochemical potential induced by O_2_ reaction. Fluctuation of the electrochemical potential induced by O_2_ reaction (background potential) may lower the sensor’s NO_2_ sensitivity. Further study on the relation between sensor’s working temperature and sensitivity is underway.

**Figure 13:**
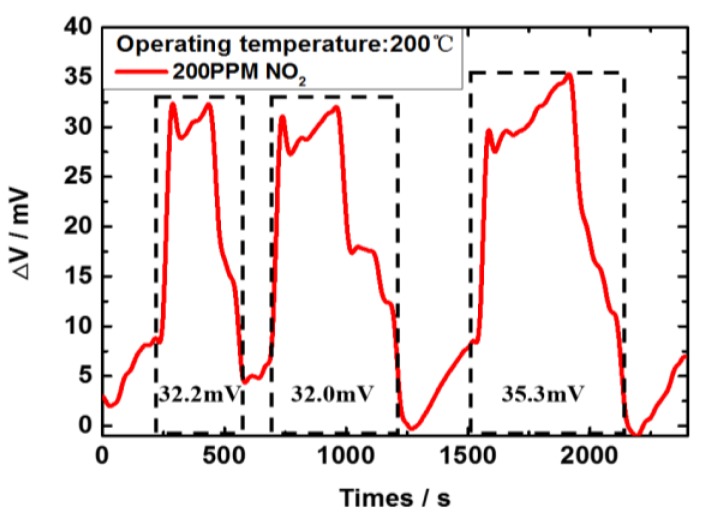
The sensor’s gas sensing response for three cycles to 200 ppm NO_2_ at a working temperature of 200 °C.

The NO_2_ sensor’s gas sensing repeatability is demonstrated in Figure 13 with alternating cycles of 200 ppm NO_2_ at the working temperature of 200 °C. Though the response curves are not identical during the testing, the average peak sensing voltage is 26.0 mV, and the difference between the peak sensing voltage to 200 ppm NO_2_ is 0.2 mV and 2.9 mV, demonstrating the sensor’s capability to detect NO_2_ emission.

**Figure 14:**
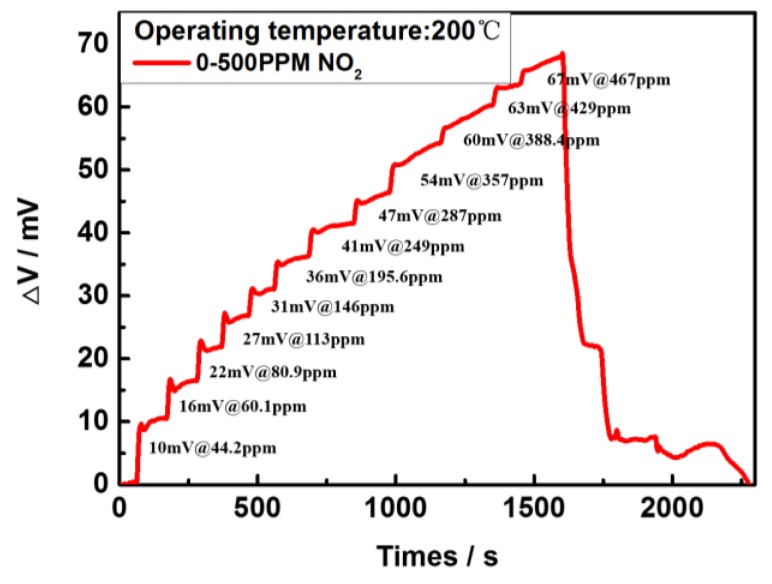
Gas sensing response of the sensor to NO_2_ in the range from 0 ppm to 500 ppm at 200 °C.

The transient response of the NO_2_ sensor tested at a concentration gradient from 0 to 500 ppm at 200 °C in 20% O_2_ environment is shown in Figure 14. The response time tends to increase with the increasing of NO_2_ concentrations. Response time (T_r_), a time required to reach 90% of the steady-state value, was dependent on the NO_2_ gas concentration. When tested at the concentration of NO_2_ in the range of 0-300 ppm, Tr is smaller than 5 s. However, the sensor’s response time (T_r_) increases with the increase of the concentration of NO_2_ from 300 to 500 ppm. The probable cause of this phenomenon is that the redox reaction on the sensing electrode surface will take some time and a higher concentration of NO_2_ gas will decrease the active reaction sites on the sensing electrode surface in a short time. Once the first batch of NO_2_ molecules finishes the absorption/desorption reaction, the NO_2_ molecules followed up could not continue the redox reaction until the previous reactants finish the desorption process. Eventually, this phenomenon would result in a larger response time of the sensor at a high concentration of NO_2_ gas. In addition, the NO_2_ sensor works at a low temperature of 200 °C with a thin thickness of sensing electrode film (0.5 µm), and its sensing performance deteriorates at higher NO_2_ concentration.

**Figure 15:**
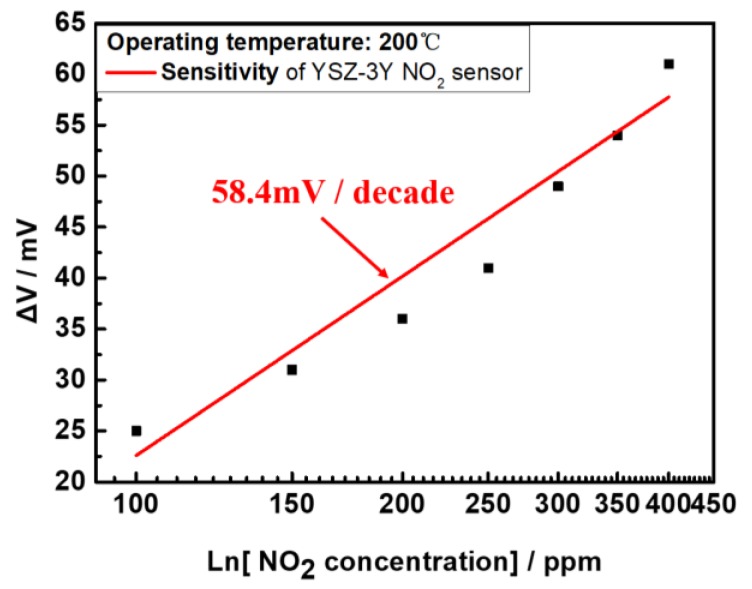
Gas sensing response to an NO_2_ concentration from 100 to 500 ppm of the flexible MPT sensor.

**Figure 16:**
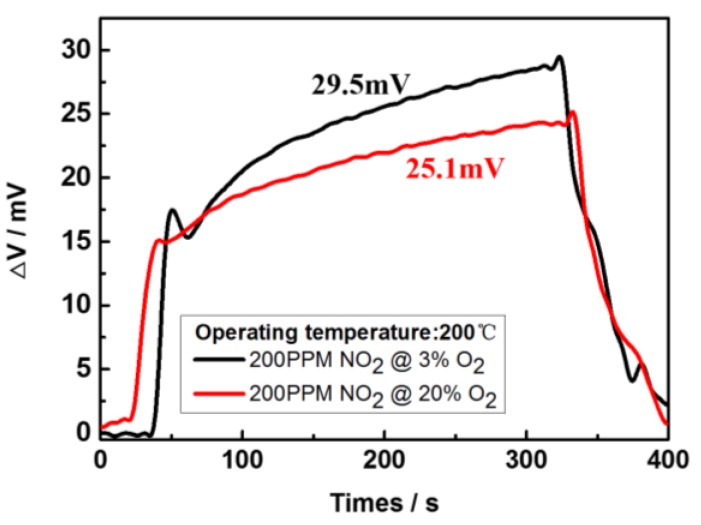
Responses of sensor to 200 ppm of NO_2_ at different concentrations of O_2_ at 200 °C.

As indicated in Figure 15, the sensing voltage of ΔV is linearly dependent on the logarithm of NO_2_ concentration in the detected range conform to MPT mechanism at a working temperature of 200 °C [30,31]. The sensitivity of the sensor was derived as 58.4 mV/decade from the linear curve’s slope, as well as a detection limit of 26.7 ppm. In addition, a variation of the oxygen concentration will affect the sensor response to NO_2_ gas, as shown in Figure 16. We compared the sensing signals at different oxygen concentration, showing a variation of the output signal of 15%, similar to the phenomena described in other literature [21,32]. 

### 3.3. Mechanical Stability of the NO_2_ Sensor

**Figure 17:**
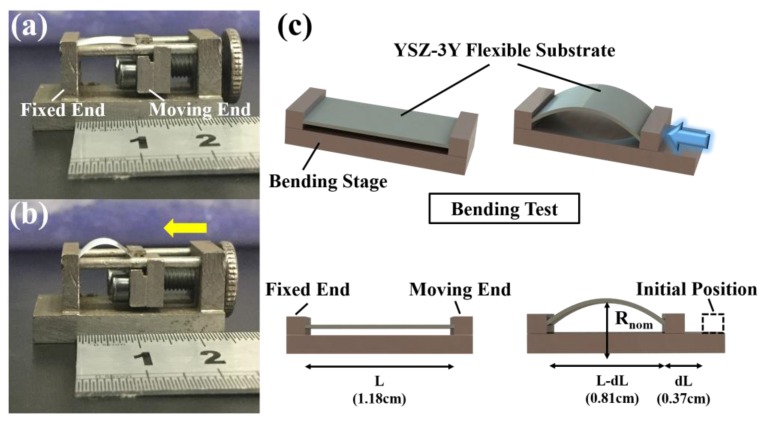
YSZ ceramic film’s mechanical property measurement: (**a**) initial state of the YSZ substrate in the bending stage; (**b**) bending state of the YSZ substrate after compression; (**c**) schematic diagram of the bending test for measuring the substrate’s bending radius and bending strain.

The ultra-thin YSZ ceramic film’s mechanical stability was characterized by measuring the film’s bending strain. As shown in Figure 17a, a YSZ substrate of 20 µm thick was put in a bending stage with a fixed end and a moving end with an initial length of 1.18 cm. Once the YSZ substrate was compressed by pushing the moving end, the substrate’s end-to-end length decreased to 0.81 cm. The substrate’s bending radius $R_{nom}$ can be calculated as 0.42 cm from Equation (1) [33,34]

(1) $$R_{nom}=\frac{L}{2\pi \sqrt{\frac{dL}{L}-\frac{\pi^{2}h_{s}^{2}}{12L^{2}}}}$$

where $h_{s}$ is the YSZ substrate’s thickness, 20 µm. L is the initial length of the YSZ substrate and dL is the decreased length of the substrate after compression. Additionally, the bending strain of the YSZ substrate $\varepsilon_{nom}$ can be calculated as 2.38 × 10^−3^ from Equation (2)

(2) $$\varepsilon_{nom}=h_{s}/\left(2R_{nom}\right)$$

**Figure 18:**
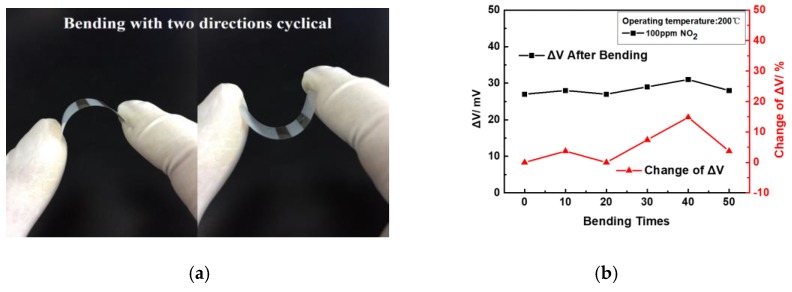
(**a**) Mechanical stability properties of sensor demonstrated in a bending fatigue experiment; (**b**) Mechanical and sensing stability performance after 10–50 times bending.

Moreover, a series of bending fatigue experiments were carried out to investigate the mechanical stability of the sensor. As shown in Figure 18 a, the flexible YSZ-based NO_2_ sensor was squeezed by two fingers to achieve a certain degree (about 30 degrees), bending to simulate the sensor’s application and performance on a non-planar substrate. The sensor was tested at 100 ppm NO_2_ concentration at 200 °C working temperature after 10, 20, 30, 40, and 50 times blending, respectively. The sensor’s performance can be characterized by ΔV, which is described in Equation (3):

(3) $$∆V=\frac{\left(∆V_{n}-∆V_{0}\right)}{∆V_{0}}\;\times \;100$$

where ΔV_n_ is sensing voltage difference of the sensor after n times of blending in the fatigue experiment. As indicated in Figure 18 b, the results show that the ΔV for the sensor at 100 ppm after 50 times was about 14.8%, which demonstrate the excellent mechanical stability of the flexible NO_2_ sensor.

It should be noted that humidity in the ambient environment [10,35] and interference from other types of gases [15], such as NO, CO, and SO_2_, are two critical parameters to affect the sensor’s performance, and related research is underway.

## 4. Conclusions

A novel design and fabrication process for flexible MPT NO_2_ sensors based on an ultra-thin YSZ ceramic film was reported. The sensor is composed of a heating electrode, a reference electrode, an NO_2_ sensing electrode on the back side and front side of an ultra-thin 3 mol% Y_2_O_3_-doped ZrO_2_ (YSZ-3Y) film 20 µm thick. The heating electrode was fabricated by a conventional lift-off process, while porous reference and sensing electrodes were fabricated by a two-step patterning method using shadow masks. Due to the ceramic film’s low thermal conductivity and thin film thickness, a low power assumption of 1025 mW was applied to obtain a working temperature of 200 °C, in an area of 8.0 mm by 6.4 mm. The sensor’s sensitivity could be achieved as 58.4 mV/decade at the working temperature of 200 °C. In addition, the flexible sensor demonstrates superior mechanical stability after bending over 50 times due to the mechanical stability of the YSZ ceramic film. These simply structured, but highly reliable flexible MPT NO_2_ sensors may lead to wide application in the automobile industry for vehicle emission systems to reduce vehicle emissions and improve fuel efficiency.

## Figures and Tables

**Table 1:** Design parameters and dimension of the sensors’ heating electrode.

Parameters	Value
3 mol% Y_2_O_3_-doped ZrO_2_ (YSZ-3Y) substrate	10 mm × 10 mm × 20 μm
Thickness of heating resistors (Å)	500 (Ti) / 4000 (Pt)
Heating electrode (μm)	94800 × 100 × 0.45
Driving voltage (V)	1–35 V
Thermal conduction coefficient (YSZ-3Y substrate) (W·m^−1^·K^−1^)	2.7
Ambient environment temperature (°C)	26 (air)
